# TaIlored ManagEment of Sleep (TIMES) for people with dementia and mild cognitive impairment in primary care in England: protocol for a feasibility cluster-randomised controlled trial

**DOI:** 10.1186/s40814-026-01791-0

**Published:** 2026-03-07

**Authors:** Jayden van Horik, Louise Allan, Aidin Aryankhesal, Niall Broomfield, Peter Greenstreet, Andrea Hilton, Anne Killett, Mizanur Khondoker, Gill Livingston, Yoon Loke, Ian Maidment, Antonieta Medina-Lara, Adam Palmer-Welsh, Joanne Reeve, Sion Scott, Lee Shepstone, Fiona C. Warren, Geoff Wong, Chris Fox

**Affiliations:** 1 University of Exeter, Exeter, England; 2 University of East Anglia, Norwich, England; 3 Ottawa Hospital Research Institute, Ottawa, Canada; 4 University of Hull, Hull, England; 5 University College London, London, England; 6 Aston University, Birmingham, England; 7 University of Leicester, Leicester, England; 8 University of Oxford, Oxford, England

**Keywords:** Sleep disturbance, Clinical trial, Complex intervention, Primary healthcare, Decision support tool, Tailored care, Dementia, Mild cognitive impairment

## Abstract

**Background:**

People living with dementia (PLWD) and mild cognitive impairment (MCI), and their family carers, often experience sleep disturbance which can impair daily living and care. There are limited options for effective long-term pharmacological management of sleep disturbance, yet recent advances in non-pharmacological approaches offer promising alternatives. TIMES is a novel, complex intervention, which aims to improve wellbeing for PLWD/MCI and their carers in primary care, by developing whole-person, tailored care plans that optimise management of sleep disturbance in context.

**Methods:**

Two-arm cluster-randomised (1:1), single-blinded, feasibility trial in 10 general practice sites in England, recruiting 64 patient–carer dyad participants (32 intervention + 32 treatment as usual). Co-primary objectives are to assess the feasibility and acceptability of conducting a subsequent definitive cluster-randomised controlled trial (cRCT) of the TIMES intervention. Secondary objectives include assessing the ability to collect data to address putative primary and secondary outcomes of a definitive cRCT. We will collect participant demographics at screening, and the following outcome measures at baseline, 9 and 15 week follow-ups: Sleep Disorders Inventory (SDI); Epworth Sleepiness Scale (ESS); Activities of Daily Living assessed with the Disability Assessment for Dementia (ADL DAD); Dementia Quality of Life Measure (DEMQOL); EQ-5D 5 level (EQ-5D-5L); ICEpop Capability measure for older people aged ≥ 65 (ICECAP-O); Neuropsychiatric Inventory Questionnaire (NPI-Q); Client Service Receipt Inventory (CSRI); Telephone Montreal Cognitive Assessment (T-MoCA); patient medical records review; patient serious adverse events (SAEs). We will conduct Process Evaluation interviews and Discrete Choice Experiments to inform refinement of the intervention content and delivery.

**Discussion:**

Our findings will inform the refinement and delivery of a subsequent definitive cRCT that tests the clinical and cost-effectiveness of the TIMES intervention compared with usual care.

**Trial registration:**

This study received approval from the Health Research Authority (HRA) and London–Harrow Research Ethics Committee (Reference: 24/LO/0123), and is sponsored by the University of Exeter (Reference: 2021–22-38). Trial registration: ISRCTN, ISRCTN54051676, registered 20 March 2024, https://www.isrctn.com/ISRCTN54051676.

## Introduction

In the UK, there are 850,000 people living with dementia (PLWD); 700,000 family carers of PLWD; and 2.4 million people living with mild cognitive impairment (MCI) [1–6]. Sleep disturbance, the increased number and duration of awakenings and increased percentage of light sleep, is common in PLWD (20–90%) and MCI (18.3–45.5%) [7–13], and has a significant impact on their day-to-day living [14–18] and family carers [19–27]. Poor management of sleep disturbance in PLWD/MCI also affects health and social care systems, due to an increase in complications from comorbidities, earlier admission to care homes, and an increased cost of healthcare [28–31].

Primary healthcare management of sleep disturbance in PLWD/MCI is difficult, as it often requires addressing multiple interacting factors (Table 1) and is rarely self-reported by PLWD/MCI [48]. PLWD/MCI and carers often request, and General Practitioners (GPs) often prescribe, licensed sleep medications, including sedative or hypnotic medications such as benzodiazepines or z-drugs [48–50]; however, there is no evidence that sedative medications are effective in the medium to long term [50, 51], and the use of hypnotics has been associated with increased incidence of fractures and strokes in PLWD [52, 53]. Cumulative sedative burden can also lead to increased daytime drowsiness and sleep disruption, which PLWD/MCI are at greater risk of developing [54–56].

**Table 1:** Multifactorial, interacting, causes of sleep disturbance in PLWD/MCI

Age-related changes, e.g. lower sleep efficiency, weakening of circadian entrainment, visual impairment, contributing to sleep–wake rhythm abnormalities [32]
Psychological disorders e.g. stress, anxiety or depression [33, 34]
Medicine side effects e.g. Donepezil (medicine licensed for dementia) and nightmares [35]
Co-morbidities e.g. arthritis, heart failure, reflux and obstructive sleep apnea [36]
Environmental factors e.g. excessive nocturnal noise or light, low daytime light exposure, uncomfortable room temperature, and nocturnal care practices [37, 38]
Activity levels e.g. reduced physical activities, social disengagement and daytime napping [39–46] Wider social determinants of health [47], including expectations and perceptions of normal sleep and de-medicalisation agenda

Non-pharmacological approaches to understand and manage sleep problems may provide alternatives to medications. Cognitive behavioral therapy for insomnia (CBT-I) is typically recommended as the first-line treatment for chronic insomnia in adults (including patients with comorbidities), and may be provided as a multicomponent treatment that includes several therapeutic strategies, such as psychoeducation (sleep hygiene) and sleep-restriction therapy [57, 58]. However, access to these treatments can be limited, and there is little evidence that supports the effective use of non-pharmacological interventions for sleep problems in PLWD [59]. Although a recent study involving a multicomponent intervention has shown improvements in the sleep of PLWD in their home, which remained beyond intervention delivery and can be implemented with minimal clinical training [60, 61].

Ideal management of sleep disturbance may be constrained by the reality of available options, for example, if medication is relied on when other non-pharmacological approaches have been tried and failed, or are not available [57, 59, 62, 63]. In weighing up the pros and cons of different sleep management approaches, clinicians must also review and manage co-morbidities that contribute to sleep disturbance, including associated medications. Patients and clinicians may then face a difficult compromise in dealing with side-effects from medications that also offer important benefits [64]. Successful management of sleep disturbance in PLWD/MCI therefore requires whole-person attention to multiple interacting contributory components, with goals and priorities of care varying both between and within individuals over time. This may be achieved by negotiating tailored sleep-management plans to meet individual needs and circumstances.

## Aims and objectives

The TIMES intervention has been co-designed, with input from PLWD/MCI, carers with lived experience, and healthcare professionals, to improve wellbeing in PLWD/MCI and carers by delivering person-centred (i.e. tailored) care that optimises sleep. This study aims to assess the feasibility and acceptability of conducting a definitive cRCT that tests the clinical and cost-effectiveness of the TIMES intervention. To achieve this, we will use quantitative and qualitative approaches to examine participant engagement, recruitment rates, retention to the trial, completeness of outcome data, intervention fidelity, and operational procedures. Additionally, we will explore the facilitators and barriers to patient recruitment to inform future trial implementation.

### Co-primary objectives

#### Objective A

Co-primary objectives are to assess the (1) feasibility (% of eligible people consenting to the study) and (2) acceptability (% of intervention participants completing all three stages of the intervention) of conducting a definitive cRCT of the TIMES intervention for PLWD/MCI, and their carers, who experience sleep disturbance.

### Secondary objectives

#### Objective B

To assess the ability to collect data to address putative primary and secondary outcomes for the definitive cRCT.

#### Objective C

To assess the ability to collect data to assess the cost-effectiveness and resource use for the definitive cRCT.

#### Objective D

To inform iterative refinement of the TIMES intervention for the definitive cRCT.

## Methods

### Design

This study is a multi-centre, two-arm, single-blinded, cluster-randomised (by site) controlled feasibility trial of the TIMES intervention. Patient participants in primary care (PLWD/MCI, with problematic sleep disturbance) and their carers (family members, friends or spouses, or paid professional carers, such as care home staff) will be recruited in pairs (dyads).

**Fig. 1:**
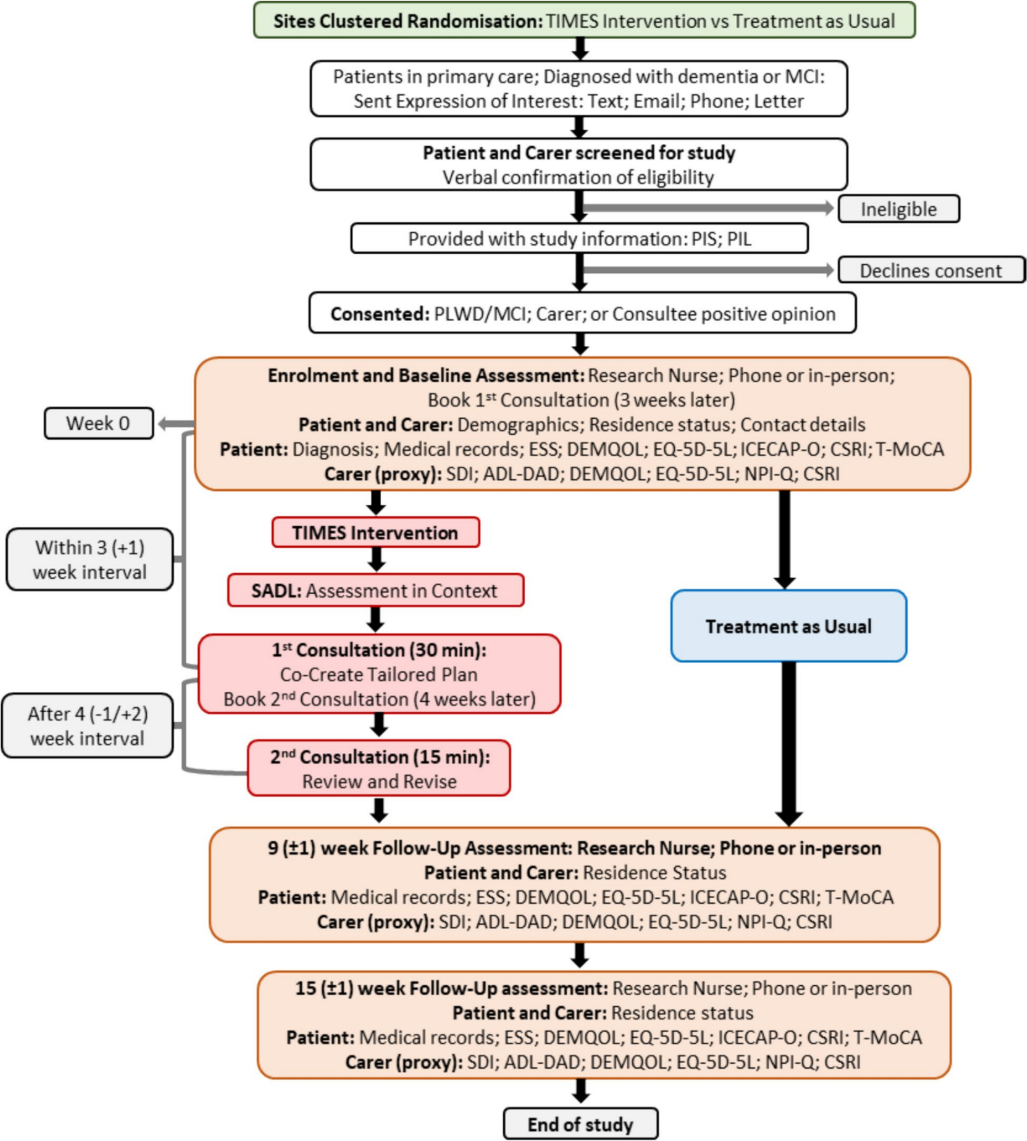
Study schema

Participant dyads recruited by sites (GP practices) that are cluster-randomised to the intervention group will be offered the TIMES intervention; participant pairs recruited by sites that are cluster-randomised to the control group will receive treatment as usual (standard care). Participant recruitment commenced in September 2024. All participants will be invited to complete baseline assessments and follow-up assessments at 9- and 15 weeks, from the date of their baseline assessment (Fig. 1). No long-term follow-up is planned.

Participants and healthcare professionals from both treatment groups will be asked to engage in an optional process evaluation interview and Discrete Choice Experiment (DCE) survey to inform an iterative revision of the intervention for a definitive cRCT.

### Screening and recruitment

We aim to recruit 64 patient–carer dyads from 10 cluster-randomised GP practices in England, UK (Intervention group = 32 dyads, Control group = 32 dyads). Potential participants will be identified from primary care practices and include patients who are diagnosed with dementia or MCI and experience sleep disturbances that are considered problematic by the person with dementia, their family, professional or family carer, or clinician. Efforts will be made through discussions with National Institute of Health and Care Research (NIHR) Regional Research Delivery Networks (RRDNs) and GP practices during site selection and recruitment to promote diversity of the participant sample, e.g. for ethnicity, socioeconomic background, and geographic location (rural/urban).

Potentially eligible patients will be identified and recruited by site staff, mail-out, text messages, emails, phone calls, and during routine consultations. Pending GP consent, patients may also be approached via a third-party data processing platform ECLIPSE (Electronic Checking Leading to Improved Prescribing/Treatment Safety & Efficiency), by text message or phone call, to ascertain expressions of interest in participating in the study. ECLIPSE is a nationally accredited NHS provider that securely manages health data for the NHS (https://www.eclipselive.org/).

Potentially eligible patient and carer participants, and consultees where applicable, will be provided with a Participant Information Sheet containing a lay summary of the study and details on how to access further information about the study. Potential patient participants must have a family carer or professional carer (i.e. care home staff). Carers will be screened and recruited to the study as a pair (dyad) with the patient participant. A blinded Research Nurse will screen potential patient and carer participants, following inclusion/exclusion criteria, and if eligible, invite them to participate in the study. Potential participants that meet the inclusion criteria will be invited to provide informed consent and enrol in the study. If the patient participant lacks capacity, a consultee will be asked to provide a favourable opinion on their behalf. A professional consultee may not also act as a carer-participant for the same dyad. Participants have the right to withdraw at any time during the study without prejudice to their care. In addition, a patient participant may be withdrawn in good faith at the request of their carer, healthcare professional, or consultee, if they feel it is within the best interest of the patient, e.g. if a patient participant shows signs of distress from participating in the study.

### Inclusion criteria

Patient participants (PLWD/MCI)

1. Aged > 18 years
2. Clinical diagnosis of dementia/MCI of any subtype and stage
3. In primary care and registered with a participating general practice
4. Residing at home or in a community care home in England
5. Has sleep problems of any type that are considered problematic by the PLWD/MCI, their family or carer.
6. Have a family or professional carer who provides support at least 1 h per week and is willing to solely assist with the completion of outcomes
7. Able to communicate in English sufficiently well to complete the outcome measures and questionnaires.
8. Has capacity to provide informed consent or has a personal or professional consultee who is able to provide favourable opinion on behalf of the PLWD/MCI

Carer participants

1. Aged > 18 years
2. Resides in England
3. Able to communicate in English sufficiently well to complete the outcome measures and questionnaires.
4. Has capacity to provide informed consent
5. Has lived or professional experience of sleep disturbance of the PLWD/MCI for whom they provide care
6. Is not already enrolled in the study with another patient participant (each carer participant can only take part once)

### Exclusion criteria

Patient participants (PLWD/MCI)

1. Deemed overburdened or has severe unstable (mental or physical) health problems (as determined by the Research Nurse or GP where appropriate)
2. Unable to communicate even with augmentative and alternative communication support
3. Does not have a family or professional carer
4. Undergoing end-of-life care
5. Planned unavailability for > 3 weeks during intervention and follow-up

Carer participants

1. Deemed overburdened or has severe unstable (mental or physical) health problems (as determined by the research nurse or GP where appropriate)
2. Diagnosis or health condition that may impair their ability to complete outcome assessments, as determined by the carer
3. Planned unavailability for > 3 weeks during intervention and follow-up

## Intervention

The TIMES intervention is a clinical assessment and management support tool, in which a tailored care plan is co-created by the patient–carer dyad and GP, subsequently reviewed by the GP, and (if necessary) revised by the patient–carer dyad and GP. While the overall intervention is a novel package—comprising GP Training, Assessment in Context (SADL), a Co-Created Tailored Plan consultation, and a Review and Revise consultation (see below)—the individual components of this complex intervention are familiar to GPs and may be delivered as part of usual care.

The intervention is comprised of the following three elements:

I. Assessment in context: patient participants will be asked to complete a one-off Sleep-related Activities of Daily Living (SADL) pre-consultation assessment. The SADL will explore and capture holistic social, health, and healthcare domains, which are associated with disruption to daily living and may be associated with problematic sleep disturbance in PLWD/MCI. The SADL will be completed after the Baseline Assessment and before the first consultation. The SADL will be used by the GP to co-create the tailored plan. Patient participants will be invited to complete the SADL in their home or care home with support from the carer participant.
II. Co-create tailored plan: a planned/pre-booked extended face-to-face consultation (30 min) between a General Practitioner (GP; not nurse practitioner), who has received Advanced Generalist intervention training relevant to the delivery of primary care in England (see below), and the patient–carer dyad will be held, to explore and improve sleep-related disruptions to daily living. This consultation should take place within 3 weeks (+ 1 week) after the Baseline Assessment is completed and review information captured in the SADL, and will occur at the participant’s GP practice, home, or care home.
  The GP will use information from (i) the SADL, (ii) the patient’s clinical record, and (iii) current guidance/best practice on condition management to inform the care planning process (using the intervention training video), alongside the discussion with the patient–carer dyad, to develop a co-created tailored care-plan for the PLWD/MCI that addresses factors affecting their sleep disturbance and its impact on their carer. The consultation will draw on best practice in Advanced Generalist Medicine. Outputs will include a written explanation of problems, intended actions, and anticipated impact of the tailored care plan for PLWD/MCI patients, which will be reviewed 4 weeks later (− 1/+ 2 weeks).
III. Review and revise: 4 weeks (–1/+ 2 weeks) after the first consultation, in which the Co-created Tailored Plan was developed, the patient–carer dyad will attend a second, pre-booked, standard in-person consultation (15 min) with their GP or, if appropriate, a relevant healthcare professional delegated by their GP, to review and if necessary revise their Tailored Care Plan. This review will be based on shared observations of whether the intended benefits/outcomes of the Tailored Care Plan have been achieved, the impact of goals and outcomes, and whether any adjustments to improve the effectiveness of the Tailored Care Plan are needed.

### Intervention training

Intervention training for GPs will be provided via pre-recorded video, and take approximately 60 min. The training video will optimise/emphasise the skills of Advanced Generalist Practice, by introducing GPs to a 3Es framework (Engagement, Enhancement, Extension) used in the consultation to co-create the tailored care-plan. Training will also address the known barriers to delivery of tailored care, by offering evidence-informed permission for AGP, and enhanced confidence in the skills and practice of AGP.

### Usual treatment

PLWD/MCI and carer participants in the Control Group will receive treatment as usual, according to appropriate NICE Guidance. This will likely vary according to individual patient needs and GP practices where the PLWD/MCI is treated. Treatment as usual will follow usual care within participating practices, underpinned by standard application of NICE pathway guidelines for dementia: assessment; diagnosis; risk assessment and management; and information [65]. Usual care for PLWD/MCI may involve sleep hygiene advice.

## Data collection

### Randomisation and blinding

#### Randomisation is at the GP practice (site) level

Ten GP practices will be randomised in batches of two sites at once, with the static list’s site number generated by the unblinded Trial Statistician (or delegate). A Trial Manager who is blinded to the static randomisation list will request the Trial Statistician to allocate treatment arms based on the order of site confirmation of capacity and capability. Requests for site confirmation of capacity and capability will be completed simultaneously in blocks of two. Five practices will be allocated to each study arm (1:1 allocation ratio), using a block design (i.e. five blocks of two practices each). Randomisation will not be stratified.

#### This is a single-blinded study

The nature of the intervention does not allow practical blinding for patient and carer participants or for intervention providers (GPs) at any level. However, recruitment, screening and collection of outcomes will be conducted by Research Nurses, who are blinded to participant treatment allocation. All analyses will be conducted by the trial statistician who is blinded to treatment allocation. Any inadvertent unblinding will be reported to an unblinded trial manager through an eCRF report on the EDC system and recorded as a deviation to protocol on an appropriate unblinded log in the Trial Management File.

### Baseline and follow-up assessments

Non-identifiable general demographic data, including age, sex, ethnicity, eligibility (or lack of), and reason for declining consent (if applicable), will be collected on screening logs for all potential participants during screening. Patient medical history, putative outcome measures for the definitive trial, and Serious Adverse Events (SAEs) will be collected at baseline and 9- and 15-week follow-ups (Table 2). All outcome measures are interviewer administered by a blinded Research Nurse, over the phone or in person. Proxy-rated (informant) questionnaires are completed by the professional/family carer participant on behalf of the PLWD/MCI participant. Approximate time to complete outcome assessments at each follow-up is estimated at 50 min for PLWD/MCI; 60 min for Carers. Participants will be informed of their treatment allocation once their baseline assessment is completed. Protocol non-compliances will be reported to an unblinded trial manager through an eCRF report on the EDC system and recorded in the Trial Management File. Any missing data fields will be queried and followed up with site staff, with explanations described in a corresponding non-compliance form. This information will be used to inform outcome selection for a definitive trial.

**Table 2:** Planned outcomes for the definitive cRCT in priority order of assessment

Clinical outcomes	Description	Time-point
Sleep Disorders Inventory (SDI) for PLWD/MCI	The putative primary outcome for a definitive trial is sleep in PLWD/MCI at 15 weeks, as assessed using the overall SDI score [66]. This subjective measure has advantages over objective sleep measures in this population, as it can be rapidly assessed during screening and avoids potential acceptability issues associated with wearable or technological devices. The overall SDI score is derived from the multiplication of frequency and severity of items 1–7, and then summed across items 1–7. A score of ≥ 4 [67] or ≥ 5 [68] on any single item (questions 1–7) is considered clinically significant. Higher scores indicate greater sleep disturbance. The SDI quantifies the (i) frequency (5-point scale), (ii) severity (4-point scale), and (iii) carer distress (6-point scale), associated with seven symptoms of sleep-disturbance that may have occurred in the past 2 weeks (see below; question 8 is not rated). It is validated for use as a proxy assessment for PLWD who reside in either domestic or care homes, and has recently been used in studies of non-pharmacological interventions in PLWD [60, 61, 69, 70]. The SDI includes carer-rated questions on whether the PLWD/MCI (1) has difficulty falling asleep; (2) gets up during the night; (3) wanders, paces or gets involved in inappropriate activities at night; (4) awakens the carer during the night; (5) awakens at night, dressing, and planning to go out, thinking that it is morning and time to start the day; (6) awakens too early in the morning; (7) sleeps excessively during the day; and (8) has other night-time behaviours that bother the carer. Approximately 10 min to complete	Baseline, 9 weeks, 15 weeks
Epworth sleepiness scale (ESS) [71]	Eight self-rated questions (scale 0–3) that assess day-time sleeping/dozing behaviour (component score range 0–24; > 10 indicates excessive sleepiness). Approximately 2–3 min to complete	Baseline, 9 weeks, 15 weeks
Activities of Daily Living (ADL): Assessed with the Disability Assessment for Dementia (DAD) [72]	Proxy-rated questionnaire consisting of 40 binary questions on the PLWD/MCI’s ability to perform basic or instrumental ADL’s in the past 2 weeks. Scores are converted to percentage (range: 0–100), with higher scores indicating greater competence in ADL. Approximately 15 min to complete	Baseline, 9 weeks, 15 weeks
Dementia Quality of Life Measure (DEMQOL) [73]	Thirty-one proxy-rated questions or twenty-eight self-rated questions (4-point scale) on quality of life, validated for PLWD at all stages and care arrangements, which can also be used to calculate utility values for estimating QALY [74]. Participants recall their experiences over their past week, including feelings, worries about their memory, everyday life, and overall quality of life. Higher scores indicate better quality of life. An improvement of at least 5/6 points for DEMQOL, and 0.02–0.05 points for DEMQOL-U, is considered clinically meaningful [75]. Approximately 10–15 min to complete	Baseline, 9 weeks, 15 weeks
EQ-5D 5 level (EQ-5D-5L) [76]	Proxy-reported by the carer, and self-rated 5 questions (5-point scale) on: Mobility; Self-care; Usual activities; pain/discomfort; and anxiety/depression. Higher scores indicate greater problems. Utility scores will be used to estimate QALYs for economic analysis. Approximately 2–3 min to complete	Baseline, 9 weeks, 15 weeks
ICEpop Capability measure for older people aged ≥ 65 (ICECAP-O) [77]	Assessment of wellbeing and quality of life in older people (ICECAP-O), with five questions (4-point scale) on: attachment (love and friendship); security (thinking about the future without concern); role (doing things that make you feel valued); enjoyment and pleasure; control (independence). Overall scores are converted into a single-index value with a range of 0–1, with zero indicating no capability (i.e. death) and one indicating full capability. Outputs will also provide an estimation of wellbeing-adjusted life years (WALYs) to inform cost-effectiveness analyses of interventions in social care. ICECAP-A and ICECAP-O instrument items correlate, scores can be calculated from each other with a good confidence, and validity of both instruments has been confirmed in the age groups 50–70 [78]. Approximately 5–10 min to complete	Baseline, 9 weeks, 15 weeks
Neuropsychiatric symptoms: Neuropsychiatric Inventory Questionnaire (NPI-Q) [79]	Twelve proxy-rated questions that assess severity (affect) of neuropsychiatric symptoms to the patient (3-point scale) and distress to the carer (5-point scale). Higher scores indicate greater neuropsychiatric changes that have occurred since the patient first began to experience memory problems. Approximately 5 min to complete	Baseline, 9 weeks, 15 weeks
Client Service Receipt Inventory (CSRI) [80]	Self- and proxy-rated health and social care service-use of the PLWD/MCI in the past 3 months for economic analysis. Questions will include the frequency of resource use in primary, community, social care, loss income from carers, etc. Approximately 10 min to complete	Baseline, 9 weeks, 15 weeks
Telephone Montreal Cognitive Assessment (T-MoCA) Mini—Version 2.1 English (MoCA 5 min/Telephone)	Self-rated non-visual cognitive assessment that can be completed over the phone and is validated for MCI and dementia, (https://mocacognition.com/paper/), assesses auditory attention, mental flexibility, verbal fluency, sentence repetition, word-list memory, and orientation to time and place, with a maximum of 15 points. Lower scores indicate greater cognitive impairment. Approximately 5 min to complete	Baseline, 9 weeks, 15 weeks

TIMES is a pragmatic intervention that has been designed to be delivered in routine clinical practice, in which a review consultation occurs at monthly intervals while modifications to intervention components are reassessed. The 15-week follow-up endpoint is therefore intended to capture relatively short-term effects.

### Process evaluation and discrete choice experiment (DCE)

Optional semi-structured interviews, with people with MCI, dementia, carers, GPs, and practice managers/admin staff who participated in the intervention, will be used to explore the process evaluation of intervention acceptability, feasibility and fidelity [81]. We will also aim to interview GPs/Practice Managers who chose not to participate or who dropped out of the study.

Interested participants will be interviewed once, and interviews will take place either near the start of the intervention, following completion of the intervention, or during follow-up, and last no more than 45 min. GPs, research nurses and administrators will also be presented with a brief, optional online survey, to share their experience on the design and delivery of the intervention and research methodology. Interview topic guides, informed by Normalisation Process Theory (NPT) [82], will be structured according to key elements to understand successful or unsuccessful implementation of a new intervention.

Analysis of process evaluation interviews and questionnaires will illuminate contextual factors that affect whether the intervention is used by PLWD/MCI, carers, and GPs as intended and whether it has had a positive impact. This will also inform assessments on the fidelity of the intervention delivery against a fidelity checklist and the intervention approach (i.e. to check that it is person-centred). The fidelity checklist will include items such as ‘was there a discussion about sleep disturbance’, participant completion of all SADL sub-elements, GP inclusion of 3E’s and goal setting in the tailored health plan, and participant attendance at GP consultations to co-develop and revise the tailored health plan. Any issues that patients or carers have in completing the SADL or attending GP consultations will also be captured in the process evaluation.

An optional DCE survey, following good practice [83–86], will be delivered to patients, carers, and GPs at Baseline and 15-week follow-up, and take up to 20 min to complete at each timepoint. Agreement to participate in the DCE will be obtained during consent (optional consent statement). Collection of DCE data from all participants who consented to participate in the DCE will be coordinated by collaborating researchers at the University of Exeter and monitored by the Trial Management Group. The DCE will be used to better understand preferences of intervention delivery. Outputs of the process evaluation and DCE will refine the programme theory and inform the design for the definitive cRCT. Participation in Process Evaluation and DCE was considered optional, as contribution from a relatively small proportion of the overall target sample size was required for meaningful interpretation of results.

### Health economic outcomes

The intended primary economic outcome measure for the definitive trial is the cost per quality adjusted life years (QALYs) [76], which will be obtained from the EQ-5D-5L, a generic measure of health related quality of life (HRQoL), and is recommended by NICE for use in health technology assessments to estimate the cost-per-QALY of interventions and to inform healthcare policy decisions across the NHS. We will also assess the feasibility of estimating cost-effectiveness for a definitive cRCT by collecting data from the DEMQOL self-rated and DEMQOL proxy, which will provide a dementia-specific measure of patient HRQoL. Health state valuations (HSVs) from carer responses to DEMQOL proxy will be derived using published and validated indices, the “DEMQOL-proxy-U” [74]. We will also assess the feasibility of collecting self- and proxy-reported data from the ICECAP-O (including adults both < 65 and ≥ 65 years), to provide a measure of patient wellbeing that is suitable for use in economic evaluation (ICECAP-O will be used instead of ICEAP-A for adults < 65 to allow comparable analysis in this study, as validity of both instruments has been confirmed in age groups 50–70 [78]. This will involve deriving wellbeing values from proxy carer responses to the ICECAP-O, using a published and validated index, which was developed to enable the cost-effectiveness of health and social care interventions to be assessed in terms of their impact on the wellbeing of older patients or care recipients, beyond the usual remit of HRQoL measures [77].

### Safety reporting

This is a low-risk intervention study (as identified with a risk assessment), which includes an older population who are expected to experience acute illness resulting in hospitalisation, development of new medical conditions and deterioration of existing medical conditions. Safety of the intervention is not an outcome measure, and the individual components of the intervention are not novel in this patient population. To reduce excessive unnecessary data collection, burden on clinical staff, and reduce the amount of sensitive patient data that is collected for research purposes, a risk-proportionate approach to the collection and reporting of safety data was taken. Only Serious Adverse Events (SAE) from patient participants will be recorded and reported. SAEs are considered as any untoward medical occurrence that results in death; is life-threatening; requires inpatient hospitalisation or prolongation of existing hospitalisation; results in persistent or significant disability/incapacity. SAEs will be recorded from the point of participant consent, or consultee declaration, to the final follow-up assessment at 15 weeks when participation in the study ends. All SAEs will be reported to Exeter CTU within 24 h of the participating site becoming aware of the event (not from the time of the event occurring). This should be done by site staff on the EDC System (REDCap). Causality of reportable SAEs will be assessed by the site PI (or authorised delegate). All SAEs which are possibly, probably or definitely related to the intervention will be categorised as ‘related’. All related serious adverse events are considered unexpected and will be categorised as RUSAEs and subject to expedited reporting to the Sponsor, DMC, PSC and lead REC. Causality of all SAEs will be assessed by the site PI (or authorised delegate).

### Patient and public involvement (PPI)

Diverse PPI groups with lived experience, coordinated by Innovations in Dementia (iD), Together in Dementia Everyday (TiDE), Chinese Wellbeing, alongside co-applicant and PPI co-lead George Rook (PLWD) and a South Asian PPI group, were involved in identifying the area of research and advising on study design from the funding application stage, through to the development and revision of all study documents and trial procedures. The TIMES intervention was developed through a series of co-designed workshops, with input from PLWD and MCI, family and professional carers with lived experience, GPs, healthcare professionals, social prescribers, and secondary care sleep clinicians [87]. Two independent PPI representatives sit on the Programme Steering Committee (PSC).

## Analysis plan

### Sample size calculation

As this is a feasibility study, the aim of the trial is not to determine the effectiveness of the intervention, but rather to estimate key feasibility parameters to inform the design of a subsequent definitive randomised trial. Consequently, no formal power calculation has been undertaken to determine the required sample size. For a total of 64 patient participants, the 95% confidence interval for follow-up will be estimated to within ± 10 percentage points, assuming a follow-up rate of 80%. This sample size will therefore be sufficient to estimate the standard deviation of outcome variables at baseline and at follow-up for each treatment group, assuming 25 participants (patient and caregiver) provide follow-up data, and assuming a medium or small effect size [88].

### Statistical analysis

A detailed Statistical Analysis Plan (SAP) will be produced by the trial statisticians (FW, PG) and the lead/senior statistician (LS), in collaboration with the Programme Management Group (PMG). The SAP will be reviewed by the Data Monitoring Committee (DMC) and by the PSC, then finalised and signed off by the trial statisticians and CI, and the independent statistician on the PSC, before completion of data collection. The SAP and Protocol will be made available upon request to the Sponsor.

Participant and carer baseline demographic and outcome data will be summarised. Specifically, continuous data will be reported as means and standard deviations, or as medians and interquartile ranges if the data appear skewed. Categorical data will be reported using numbers and percentages. A CONSORT flow diagram will be produced to illustrate the flow of participants through the trial. Specifically, the number of participants approached, eligible, consented and recruited, and assessed at baseline, and at 9- and 15-week follow-up, will be illustrated, along with the number of participants withdrawn or lost to follow-up between each data collection time point. The reasons for ineligibility, eligible participants not being recruited, and for withdrawal post-consent, will also be presented where available. To participate in the trial, both the patient and their carer need to be eligible and willing to consent. As a result, we will also present the ineligibility and unwillingness to participate figures, broken down by patient and carer.

An objective of this feasibility study is to assess the completeness of the primary and secondary outcomes proposed for the definitive trial. Summary statistics, including mean differences and CIs, for each continuous outcome at baseline and at 9- and 15-week follow-up will be presented by treatment group. In addition, mixed linear regression models will be performed to produce a between-group mean difference and associated 95% confidence interval at 15-week follow-up for continuous outcomes. Linear mixed regression models will be performed with adjustment for baseline values and centre, i.e. region, and will include a random effect on cluster (practice), with a small sample correction to accommodate for the small number of clusters sampled. For SDI (the putative primary outcome for the definitive trial), we will derive a binary variable at all time-points to indicate whether or not the patient participant had at least one (of seven) symptoms with scores (frequency × severity) of ≥ 4 [67]; this binary variable will be reported descriptively by treatment group. All analyses will follow the treatment policy approach, i.e. participants will be analysed according to their randomised group irrespective of treatment actually received. All analyses will use observed data only. If individual items within a patient-reported outcome measure (questionnaire) are missing, these may be imputed in line with instrument-specific published guidance or scoring instructions, or other method as agreed by the trial team. However, no between-person imputation (e.g. multiple imputation for dropouts) is planned.

As this is a feasibility study, no inferential subgroup analyses will be undertaken. However, it is of interest to explore the feasibility outcomes separately for participants with dementia and for those with MCI, as well as participants with family and professional carers. As a result, the summary descriptive statistics for each putative outcome measure will be presented within treatment group for participants with PLWD and participants with MCI separately, and for participants with family carers and those with professional carers separately. All analyses of continuous outcome measures will be adjusted for baseline values. There will be no sensitivity analyses with further adjustment for participant characteristics, even if unbalanced by treatment group, due to the feasibility nature of the trial. There are no planned interim analyses or criteria for early termination of the trial. No sensitivity analyses using imputed data for those participants who do not have observed outcome data will be performed. Further detail will be provided in the SAP. The degree of missing data for each outcome at each time point will be reported to inform the design of a subsequent definitive trial.

Qualitative interviews and surveys for process evaluation will be analysed using open coding techniques, leading to thematic descriptions of the combined datasets. The analysis will reflect the core constructs of Normalisation Process Theory (NPT), adding the patient/carer’s perspective, which will enable findings, coherently embedded in the NPT framework, to inform future implementation.

### Health economic analysis

We will describe mean costs and mean effects, presenting disaggregated costs and consequences. Methods will be designed for capturing: (i) the resources required to deliver the intervention; (ii) health, social and wider care service resource use; (iii) health economic outcomes relating to HRQoL and capability wellbeing. These findings will inform the development of future economic evaluation to be undertaken alongside the definitive cRCT to: (i) establish the resources required to provide the intervention, (ii) estimate intervention costs, and (iii) conduct a full cost-economic analysis (CEA). The intervention costing and CEA, based on within-trial data collection, will be undertaken against a primary perspective of the NHS/Social Care, with participants and broader societal perspectives considered in sensitivity analyses. The future CEA will synthesise cost and outcome data to present an incremental cost-effectiveness ratio (ICER) for the primary economic endpoint of policy relevance, including cost per quality-adjusted life-year (QALY).

Descriptive statistics will be presented for dementia-specific HSVs and wellbeing values at each assessment point (mean, standard deviation, range) and for QALYs and WALYs over the 15-week follow-up period, calculated using the standard AUC approach. We will use an intention-to-treat analysis, taking the previously mentioned NHS and social care perspective. No subgroup analysis will be conducted as this is a feasibility trial and not powered for inferential analyses. As this is a feasibility trial, we will report descriptive data only for each trial arm, with confidence intervals for between-group comparisons. No *p*-values will be reported. Primary analysis will be based on complete case data, in which each individual analysis will be based on observed (i.e. not imputed) data from participants with completed fields that are required for each respective analysis. Losses to follow-up at data collection points will be presented for both primary and secondary outcomes. Missing outcome data will also be discussed, along with its causes. Outputs from the feasibility study will follow CHEERS reporting conventions [89].

## Feasibility outcomes

Primary and secondary objectives will be assessed according to feasibility outcome measures (Table 3). Assessment of the ability to collect these data will be used to improve the design and delivery of a definitive cRCT of the TIMES intervention.

**Table 3:** Feasibility objectives matched to feasibility outcome measures and Time-points

Feasibility objectives	Feasibility outcome measures	Time-point(s) of evaluation
Primary objective		
Objective A: the co-primary objective is to assess the (1) feasibility (% of eligible people consenting to the study) and (2) acceptability (% of intervention participants completing all three stages of the intervention) of conducting a definitive randomised controlled trial of the TIMES intervention for PLWD/MCI, and their carers, who experience problematic sleep disturbance	The proportion of eligible people who consent to participate in the study The proportion of consented participants who remain in the study and provide valid outcome data for clinical and health-economic measures, including the putative primary outcome for a definitive trial at 9- and 15-week follow-ups The acceptability of the intervention as assessed through process evaluation	Recruitment Post intervention 15-week follow-up
Secondary objectives		
Objective B: To assess the ability to collect data to address primary and secondary outcomes for the definitive RCT	The proportion of PLWD/MCI in primary care with problematic sleep disturbance (per 1000 registered patients) The proportion of PLWD/MCI in primary care, with problematic sleep disturbance and a carer (per 1000 registered patients) The proportion of PLWD/MCI in primary care, with problematic sleep disturbance and a carer who meet the eligibility criteria (per 1000 registered patients) The proportion of consented participants in the intervention arm who start the intervention The proportion of consented participants in the intervention arm who complete all three stages of the intervention The proportion of consented participants who remain in the study until final follow-up at 15 weeks The proportion of consented participants who remain in the study and provide valid outcome data for clinical and health-economic measures including the putative primary outcome for a definitive trial at 9- and 15-week follow-ups Mean and standard deviation, plus between group mean difference with 95% confidence interval and intraclass correlation coefficient (ICC) for the proposed primary outcome of the definitive trial (and any proposed outcomes that may be key secondary outcomes for which a power calculation is performed), used to verify or revise the proposed primary outcome and sample size calculation for the definitive RCT	Recruitment Recruitment Recruitment 9-week follow-up 9-week follow-up Post intervention 15-week follow-up 15-week follow-up
Objective C: To assess the ability to collect data to assess the cost effectiveness and resource use for the definitive RCT	The proportion of consented participants who remain in the study and provide valid outcome data for clinical and health-economic measures including the putative primary outcome for a definitive trial at 9- and 15-week follow-ups	9- and 15-week follow-up
Objective D: To inform iterative refinement of the TIMES intervention for the definitive RCT	The acceptability of the intervention assessed during the process evaluation	Post intervention

## Progression criteria

Criteria for determining whether to proceed to a definitive trial have been reviewed and agreed by an independent Programme Steering Committee (PSC) (Table 4). Any ambiguity in meeting the overall progression criteria will be made by the PSC in conjunction with the Funder.

**Table 4:** Summary of progression criteria to definitive trial

	Definite Stop, i.e. abort definitive cRCT	Review required, i.e. PSC to determine progression to definitive cRCT	Definite Go i.e. proceed to definitive cRCT
Feasibility: recruitment rate	< 25% of invited eligible people consenting to participate	25–79% of eligible people consenting to participate	≥ 80% of eligible people consenting to participate
% of target sample size recruited	< 50%	50–99%	100%
Acceptability: retention to trial	< 25% of participants complete all three stages of the intervention	25–89% of intervention participants complete all three stages of the intervention	≥ 90% of intervention participants complete all three stages of the intervention
Completeness of outcome data	< 25% of participants complete key outcome data at 15-week follow-up	25–89% of participants complete key outcome data at 15-week follow-up	≥ 90% of participants complete key outcome data at 15-week follow-up
Process evaluation	Evidence that the intervention cannot be delivered with fidelity and that it is not acceptable to participants and professionals	Inconclusive evidence that the intervention can be delivered with fidelity and that it is acceptable to participants and professionals	Evidence that the intervention can be delivered with fidelity and that it is acceptable to participants and professionals

### Study timeline

Twenty months total: 9 months set-up; 8 months participant recruitment, trial delivery and data collection; 3 months analysis and intervention refinement.

### Risks

The TIMES intervention aims to optimise the delivery of existing elements of healthcare, including medication, but is a low-risk, non-pharmacological intervention and is hence categorised as Type A = no higher than the risk of standard medical care [90].

### Protocol date and version identifier

This manuscript was based on the final version of the study Protocol version 11 24-Feb-2025 (unpublished). Amendments to trial documents will be managed via IRAS and communicated to sites and Regional Research Development Networks (RRDNs).

### Data protection and patient confidentiality

Study data will be pseudonymised. Any data will be reported anonymously so that it will not be possible to identify any individual taking part in the study. Data will be collated, managed, stored, and processed by the UKCRC registered Exeter Clinical Trials Unit (ExeCTU), via a secure online study EDC system (REDCap Academic) [91, 92], following UK GDPR, Data Protection Act 2018, and ICH GCP E6 R2, and the secure NHS-England-validated data processing system ECLIPSE (Prescribing Services Ltd.: Company number 05913240; Data Protection Registration number Z2536678; Info Security ISO 27001; Environmental 14,001; and Quality Management ISO 9001). The trial Data Management Plan can be accessed upon request to the Sponsor.

### Dissemination plan

We aim to publish findings within 24 months of study completion, regardless of outcome, in open-access peer-reviewed scientific and clinical journals, and via presentations at local, national, and international meetings, in line with NIHR guidelines. Outcome papers will adhere to CONSORT guidelines. We will work with PPI groups to provide a lay-accessible summary of the results to all study participants. Participants will be asked to provide their contact method preferences so that they receive the results in a format of their choice.

### Role of sponsor and funder

The University of Exeter is the sponsor for this study. The Sponsor has had input into the design of the study but overall responsibility for the design lies with the Chief Investigator (CI) and co-applicants. The Sponsor is responsible for authorising the initial submission to the Research Ethics Committee (REC) and Health Research Authority (HRA) and subsequent amendments, ensuring appropriate agreements and indemnity arrangements are in place, overseeing the conduct of the study and ensuring it adheres to the relevant principles of Good Clinical Practice (GCP) and the UK Policy Framework for Health and Social Care Research and for archiving at the end of the study. The sponsor is not responsible for, and has no involvement in, the data analysis or interpretation, or for writing manuscripts.

The NIHR as funder is responsible for providing funds to cover the agreed research costs as part of a programme grant. The funder is not responsible for, and has no involvement in, data analysis or interpretation, or for writing manuscripts.

Sponsor contact details: Research Ethics and Governance Office, University of Exeter, The Innovation Centre, Rennes Drive, Exeter, EX4 4RN. Email: res-sponsor@exeter.ac.uk.

## Roles of trial management group and independent oversight committees

### Programme steering committee (PSC)

Chair: Simon Coulton, Professor of Health Services Research, University of Kent.

The PSC includes an independent chairperson with expert knowledge in the subject area, a minimum of two independent professional members, and a minimum of one independent lay representative. PSC members, excluding the Programme/Trial Manager and at least one trial statistician, will be blinded to trial arms, provide advice, and provide support and recommendations to the Funder and PMG/TMG on the overall conduct of the trial. The PSC will meet at least annually throughout the duration of the trial. The specific roles and responsibilities of the PSC are documented in the PSC charter, available upon request to the Sponsor. The PSC will act as a Trial Steering Committee (TSC).

### Data monitoring committee (DMC)

Chair: Phyo Myint, Professor of Old Age Medicine (Clinical), University of Aberdeen.

The DMC includes an independent chairperson with expert knowledge in the subject area, and two independent professional members: one statistician and one clinician. DMC members will be unblinded to trial arms and provide advice, support and recommendations to the PSC on trial non-compliances, data integrity, data completeness, and safety reporting. The DMC will meet at least annually throughout the duration of the trial. The specific roles and responsibilities of the DMC are documented in the DMC charter, available upon request to the Sponsor.

#### Programme Management Group (PMG)

The PMG includes the chief investigator, programme collaborators and co-applicants, statisticians, qualitative researchers, health economists, Patient and Public Involvement (PPI) representatives, Sponsor representatives, ExeCTU data and trial management staff, and the programme/trial manager. The PMG will meet at least quarterly to monitor safety, key performance indicators and discuss and resolve emerging issues.

#### Trial management group (TMG)

The TMG includes the chief investigator, key programme collaborators and co-applicants, including statisticians, qualitative researchers, health economists, Sponsor representative, ExeCTU data and trial management staff, and the programme/trial manager. The TMG is responsible for producing the protocol, participant-facing materials and the statistical analysis plan (SAP), and will obtain relevant approvals from the relevant Research Ethics Committee (REC) and the Health Research Authority (HRA), coordinate with primary care practices to set up sites, and ensure the study is conducted according to the relevant principles of GCP and the UK Policy Framework for Health and Social Care. The TMG will meet at least monthly to manage the day-to-day running of the study and to monitor safety, key performance indicators, including trial adherence, and discuss and resolve emerging issues. Members of the TMG will analyse data, interpret analyses, report to the funder, write manuscripts for publication in peer-reviewed journals and disseminate findings at conferences and similar events.

### Indemnity

The University of Exeter, as Sponsor of the trial, holds clinical trial insurance to provide for the payment of compensation to research participants arising from injury or illness arising from the study where there is no legal liability. If an injury arises as a result of how the trial has been set up, insurance cover is provided by the University of Exeter’s clinical trials policy on a non-negligence basis. If an injury is caused by an NHS member of staff whilst carrying out any medical intervention to the participant in the study, the participant will need to pursue a claim via the NHS indemnity scheme. GP practices selected to participate in this study shall provide clinical negligence insurance cover for harm caused by their employees. A copy of the relevant insurance policy or summary shall be provided to the University of Exeter, upon request.

### End of study and post-trial care

The study will end for a participant after their 15-week assessment is complete. After this point, patient participants will continue to receive standard NHS care, with no special arrangements made in relation to the study. The patient and carer may discuss continuation of their tailored care plan with their GP, as part of their ongoing usual care, if appropriate.

## Data Availability

The dataset supporting the conclusions of this article will be made available in the University of Exeter’s Open Research Exeter repository (https://ore.exeter.ac.uk/repository/).
